# Changes in brain structure in subjects with resistance to thyroid hormone due to *THRB* mutations

**DOI:** 10.1186/s13044-023-00176-2

**Published:** 2023-08-17

**Authors:** Berenike Rogge, Marcus Heldmann, Krishna Chatterjee, Carla Moran, Martin Göttlich, Jan Uter, Tobias A. Wagner-Altendorf, Julia Steinhardt, Georg Brabant, Thomas F. Münte, Anna Cirkel

**Affiliations:** 1 Department of Neurology, University of Lübeck, Ratzeburger Allee 160, 23538 Lübeck, Germany; 2 Department of Psychology II, University of Lübeck, Lübeck, Germany; 3 Wellcome-MRC Institute of Metabolic Science, University of Cambridge, Cambridge, UK; 4 Beacon Hospital, Dublin, Ireland; 5 St Vincent’s University Hospital, Dublin, Ireland; 6 School of Medicine, University College Dublin, Dublin, Ireland; 7 Department of Internal Medicine I, University of Lübeck, Lübeck, Germany

**Keywords:** Resistance to thyroid hormone, voxel based morphometry, Diffusion tensor imaging, Thyroid hormone receptor beta

## Abstract

**Background:**

Being critical for brain development and neurocognitive function thyroid hormones may have an effect on behaviour and brain structure. Our exploratory study aimed to delineate the influence of mutations in the thyroid hormone receptor (TR) ß gene on brain structure.

**Methods:**

High-resolution 3D T1-weighted images were acquired in 21 patients with a resistance to thyroid hormone ß (RTHß) in comparison to 21 healthy matched-controls. Changes in grey and white matter, as well as cortical thickness were evaluated using voxel-based morphometry (VBM) and diffusion tensor imaging (DTI).

**Results:**

RTHß patients showed elevated circulating fT4 & fT3 with normal TSH concentrations, whereas controls showed normal thyroid hormone levels. RTHß patients revealed significantly higher scores in a self-rating questionnaire for attention deficit hyperactivity disorder (ADHD). Imaging revealed alterations of the corticospinal tract, increased cortical thickness in bilateral superior parietal cortex and decreased grey matter volume in bilateral inferior temporal cortex and thalamus.

**Conclusion:**

RTHb patients exhibited structural changes in multiple brain areas. Whether these structural changes are causally linked to the abnormal behavioral profile of RTHß which is similar to ADHD, remains to be determined.

## Introduction

Thyroid hormone levels and transporter proteins influence the development of the human brain. Brain development is mediated by thyroid hormone action [1]. Irregularities in balance of thyroid hormones at precise developmental timings can lead to somatic and cognitive changes [2]. We have previously shown that a period of only several weeks duration of induced hyper- or hypothyroid states influences the function and structure of the brain, without significant measurable somatic changes in parameters such as heartrate or blood pressure [3–6]. Moreover, hypothyroidism during adulthood induces morphological changes in the brain [7].

Thyroid hormones regulate developmental and physiological processes, acting via nuclear, thyroid hormone receptors (TRa, TRb), to alter transcription of target genes. Mutations in receptor genes (*THRB and THRA*), cause syndromes of Resistance to Thyroid hormone (RTHb, RTHa) [8, 9], whose phenotypes differ due to the differential expression of TR isoforms in tissues (TRα1: central nervous system, myocardium, skeletal muscle, bone and gastrointestinal tract; TRβ1: liver, kidney; TRβ2: hypothalamus, pituitary, cochlea, retina) [10].

RTHβ, due to heterozygous mutations in *THRB*, is a relatively uncommon disorder with over 800 families with 200 different receptor mutations being recorded to date [11]. Due to impaired function of the TRβ2 isoform expressed in the hypothalamus and pituitary [12], normal negative feedback regulation of TSH by thyroid hormones is perturbed, resulting in raised circulating free thyroid hormones (fT4, fT3) with non-suppressed TSH concentrations [10]. Due to differential distribution of TR subtypes, RTHβ patients exhibit symptoms reflecting hypo- and hyperthyroid states of specific tissues [10]. Typical phenotypes in RTHβ include goiter, resting tachycardia, recurrent ear infections in childhood causing hearing loss, altered photoreceptor function and attention-deficit hyperactivity disorder (ADHD) [13–15]. Indeed, previous studies suggest that ADHD is the main neurocognitive abnormality in RTHβ, with approximately half of RTHβ patients exhibiting an ADHD-like phenotype [16–19].

The differential tissue distribution of TRs suggests that RTHß patients might show abnormalities in brain structure, which, in turn, might be related to behavioural changes. Accordingly, in this study, changes in grey matter volume using voxel based morphometry, were analyzed [20, 21]. Previous studies of patients in hyper- or hypothyroid states [3, 7, 22, 23], have revealed structural changes, suggesting that this method would also reveal changes in RTHß. Measurement of cortical thickness has also highlighted structural changes in thyroid disease [24, 25].

An earlier publication had suggested that male RTHb patients exhibit multiple Heschl’s transverse gyri in the primary auditory cortex [26], so we sought to verify these findings in the current study.

Thyroid hormones have been shown to regulate myelination of neurons [1]. Such changes in myelination in brain white matter are reflected in different parameters gleaned from diffusion tensor imaging (DTI). For example, reductions of fractional anisotropy (FA) have been found in hypothyroid patients in the corticospinal tract, the posterior limb of the internal capsule, uncinate fasciculus, and inferior longitudinal fasciculus [27]. Our exploratory study aimed to delineate the influence of mutations in the thyroid hormone receptor (TR) ß gene on brain structure.

## Materials and methods

### Subjects

In total forty-two subjects were recruited; twenty-one RTHβ subjects (mean age 39 y, SD 15.0, 12 women) were matched with 21 healthy controls (mean age 38 y, SD 14.0, 12 women, from Lübeck, Germany). The participants in this study are unselected cases of RTHb, diagnosed in Cambridge following referral to this centre for investigation of discordant thyroid function (raised thyroid hormones, non-suppressed TSH). The investigation of all participants took place at the University Medical Centre Schleswig-Holstein, Campus Lübeck, Germany. The patients carried the following heterozygous TRß mutations: R320H (n = 5), R438H (n = 4), R429Q (n = 3), R383C (n = 2), M310V (n = 1), G345C (n = 1), P453S (n = 1), R243W (n = 1), T277I (n = 1), R338W (n = 1), E460K (n = 1). Mutations were maternally (n = 12) or paternally (n = 3) inherited or occurred *de novo* (n = 6). Medication in single patients included thyroxine for coincident autoimmune hypothyroidism (n = 1), propranolol in reduced dosage at initial referral, alfacalcidol for postsurgical hypoparathyroidism and atenolol for high blood pressure. All patients were screened for general health, drug abuse and medical comorbidities, with evaluation of thyroid status (TSH, fT4, fT3), and fasting lipid profiles (Total, LDL and HDL cholesterol). All patients were examined by an endocrinologist. Their structural brain images were evaluated and approved to be normal by a neuroradiologist. All subjects were right-handed.

Blood parameters were analysed on serum (transported at minus 80) in Cambridge with TSH, fT3 and fT4 being measured by Advia Centaur (Siemens) as described previously [28]. The reference ranges of hormone measurements were as follows: fT3 3.5–6.5 pmol/l, fT4 10–19.8 pmol/l and TSH 0.35–5.5 mU/l.

### Attention deficit analysis

We used the *Adult ADHD Self-Report Scale* (ASRS-v1.1) [29], composed of 18 questions describing typical symptoms of ADHD consistent with the Diagnostic and Statistical Manual of Mental Disorders (DSM) criteria. The test asks for typical symptoms (i.e. deficits in attention, concentration), impairments (i.e. at work, school or in family settings) and history (i.e. were the symptoms also present in childhood). Additionally, the *ADHD Rating Scale-IV* was used, consisting of two subscales including 9 items scaling inattention and 9 items regarding hyperactivity impulsivity [29]. To test for group differences independent t-test per *ADHD Rating Scale-IV* subscales will be used.

### MRI data acquisition and analysis

Structural MR imaging was performed at the CBBM Core Facility Magnetic Resonance Imaging using a 3-T Siemens Magnetom Skyra scanner equipped with a 64-channel head-coil. Structural images of the whole brain were recorded using a 3D T1-weighted MP-RAGE sequences were acquired (TR = 1900 ms; TE = 2.44 ms; TI = 900 ms; flip angle 9°; 1 × 1 × 1 mm^3^ resolution; 192 × 256 × 256 mm^3^ field of view; acquisition time 4.5 min). Diffusion-weighted data were recorded using a 64-direction DTI sequence (Single-Shot EPI sequence, 70 slices, TR = 6100 ms, TE = 116 ms, FOV 244 × 244 mm^2^, voxel size 1 × 1 × 2 mm^3^, flip angle 90, b-value 1500 s/mm^2^, one b_0_ (without diffusion weighting) image at the beginning and 4 b-zero images at the end of the sequence). Analysis was corrected for age and gender.

#### Diffusion tensor imaging

Diffusion tensor imaging (DTI) is an imaging technique enabling to non-invasively measure white matter changes in the central nervous system. Preprocessing including eddy correction and rotation of the vector definitions was performed using the FMRIB Software Library [30]. The resulting tensor images were transformed to DTI-ToolKit data format (http://www.nitrc.org/projects/dtitk/) and registered to the IIT tensor template provided by the IIT atlas [31] combining rigid, affine, and diffeomorphic registration steps. Based on the spatially normalized tensor images DTI-ToolKit was also taken to calculate individual FA maps. To test for group differences SPM12 toolbox was used to perform a two-sample t-test with age as covariate. Statistic images were assessed for cluster-wise significance using a cluster-defining threshold of P = 0.001; the 0.05 FWE-corrected critical cluster size was 275.

#### Voxel based morphometry

Voxel-based morphometry (VBM) is a technique to analyses structural changes of the brains grey matter using T1-weighted MR images. It measures differences of grey matter by a voxel-wise comparison of multiple brain images. VBM analysis was evaluated in the whole brain, carried out using Statistical Parametric Mapping 12b (SPM, http://www.fil.ion.ucl.ac.uk/spm) and Computational Anatomy Toolbox (http://www.neuro.uni-jena.de/cat/; version 12.6, 1445) in Matlab R2019b. Preprocessing of the data comprised tissue segmentation and spatial registration using DARTEL, removal of inhomogeneities and noise, global intensity normalization and spatial smoothing (12 mm FWHM Gaussian Kernel). Total intracranial volume (TIV) was also calculated. After preprocessing a two-sample t-test was computed as group statistic for every voxel, whereby age and intracranial volume were considered as confounding factors. Since we found no significant differences when applying a correction for multiple testing, we considered the results also at an uncorrected p-value of 0.001, which is a common method to explore patient data. Due to an increase of the alpha error it has to be acknowledged, though, that this approach may produce false positive results.

#### Cortical thickness

Cortical thickness analysis measures the width of grey matter in the human cortex. The analysis of cortical thickness was also performed with SPM12 and the CAT toolbox using the algorithm described by Dahnke et al. [32]. Based on the VBM preprocessing steps the central surface and the cortical thickness was estimated using a projection based thickness approach [32]. Initial surface reconstruction was followed by repair of topological defects and surface refinement resulting in the final central mesh [33]. For statistical analysis we followed the program’s recommendation using a 15 mm FWHM Gaussian kernel for spatial smoothing. We calculated a two-sample t-test with age as covariate. To correct for multiple comparisons at cluster level = 633 a threshold of p = 0.05 (FWEc) and a cluster defining threshold of p = 0.001 was applied.

#### Relationship brain structure and attention deficit

To test for a correlational relationship between structural changes and Attention deficit test scores regions of interest (ROIs) will be defined by the clusters resulting from the group comparisons. Mean FA and VBM scores extracted from these clusters will be correlated with test scores which also show a significant difference between groups. Since CAT toolbox does not allow for the individual definition of ROIs mean values will be extracted from the atlas definition in which the significant group difference was observed. The atlas definition used here was the Desikan-Killiany Atlas. Correlations were calculated using spearman’s rho. Since the correlational analysis was exploratory we did not correct for multiple comparisons.

#### Analysis of Heschl’s gyri

The sizes of Heschl’s gyri were measured manually, the brain region was selected by specialists voxel by voxel. The program mricron (https://www.nitrc.org/projects/mricron [34] was used to define the region layer-by-layer with manual tracing using a mouse-guided cursor. Heschl’s gyri analysis was performed in a blinded fashion, first in independent sessions, followed by a subsequent combined session by two different examiners (one neurologist, one neuroscientist). In line with previous reports number of Heschl’s gyri was classified into typical (one gyrus) and atypical (multiple gyri) [26, 35, 36]. Prior to performing the analyses, the examiners agreed to the procedures during a joint session using sample brain images. Differences between the number of typical and atypical Heschl’s gyri were statistically tested using a chi squared test.

## Results

### Circulating thyroid hormone concentrations

Mean TSH was shown to be within the normal range in both RTHβ patients and control subjects. Both fT4 (RTHβ: Mean 28.4 pmol/L, SD 5.5 pmol/L. Controls: Mean: 14.6 pmol/L, 1.6 pmol/L. P < 0.001, two-sample t-test) and fT3 (RTHβ: Mean 8.6 pmol/L, SD 1.6 pmol/L. Controls: 5.1 pmol/L, SD 0.5 pmol/L. P < 0.001, two-sample t-test) concentrations were significantly elevated in RTHβ patients, but were within the normal range in control subjects.

### Clinical symptoms

All subjects were examined by an endocrinologist and a neurologist with additional training in psychiatry. Out of the 21 RTHβ patients, one showed tachycardia, whereas eleven reported occasional palpitations. In the clinical history, 9 patients reported difficulties in concentrating and 12 reported anxiety episodes. Other signs and symptoms of hyperthyroidism (increased perspiration, peripheral tremor, proximal myopathy, increased stool frequency, weight loss, changes in menstrual cycle) were not present. None of the patients exhibited features of hypothyroidism (e.g. cold intolerance, constipation, weight gain, dry skin, hair loss, bradycardia, delayed relaxation of tendon reflexes, carpal tunnel syndrome).

### Attention deficit analysis

The self-rating questionnaires for ADHD I and II revealed significantly higher scores in the RTHβ group (ADHD I mean = 95.7 (9.1), ADHD II mean = 36.1 (2.3)) in comparison to controls (ADHD I mean = 60.6 (4.8), ADHD II mean = 24.1 (1.7); ADHD I: RTHβ vs. controls t(36)=|3.31|, p = 0.002; ADHD II: RTHβ vs. controls t(36)=|4.16|, p = 0.0018).

### Imaging results

**Fig. 1:**
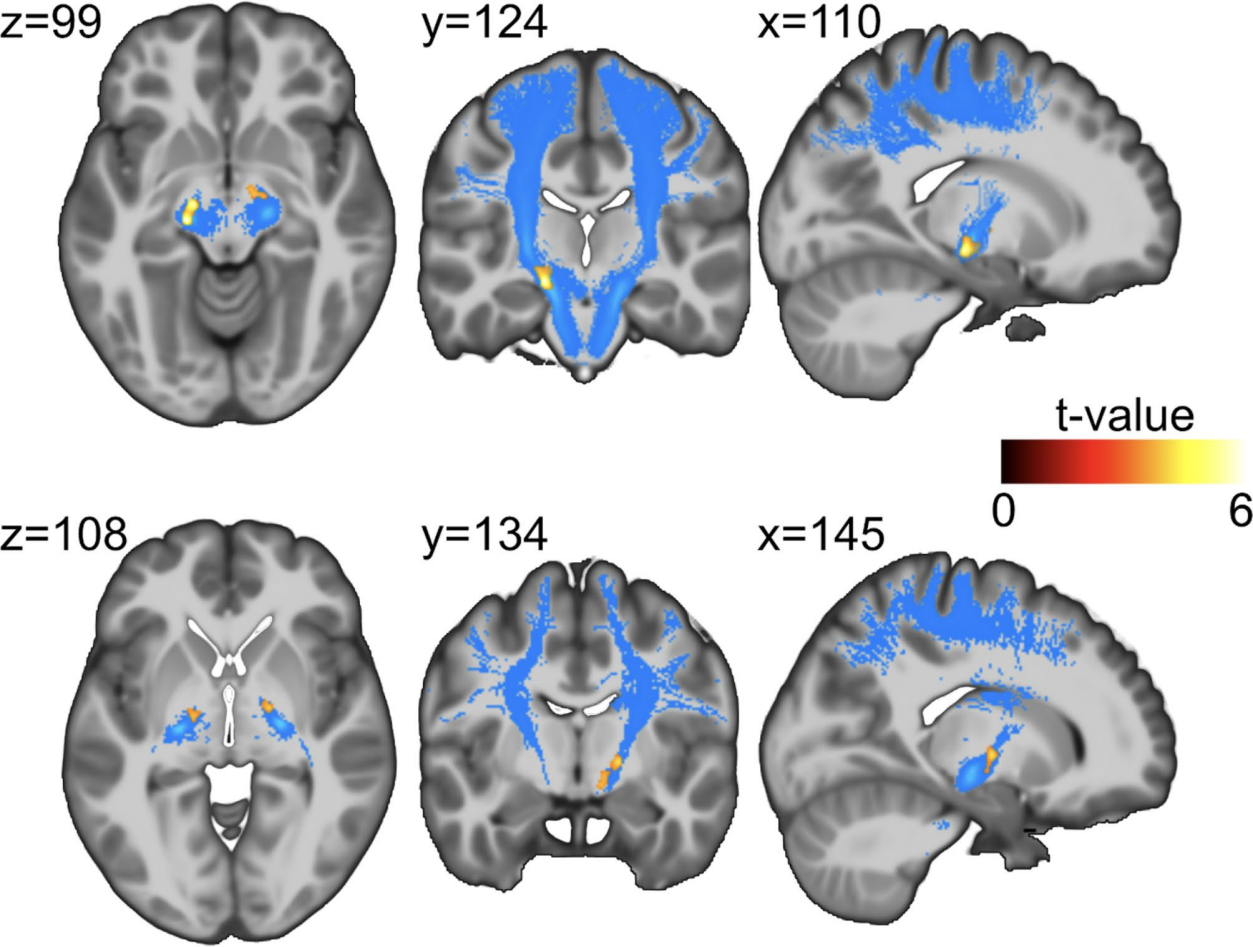
Significant FA differences between healthy controls and RTHß patients displayed onto the average T1 image in IIT atlas standard space (voxel size 1 mm). Top row shows the significant cluster of the comparison healthy controls > RTHß in the left hemisphere, bottom row the significant cluster for the same comparison in the right hemisphere (FWEc 0.05, k = 275). Additionally depicted in blue is the corticospinal tract according to the IIT atlas definition

**Fig. 2:**
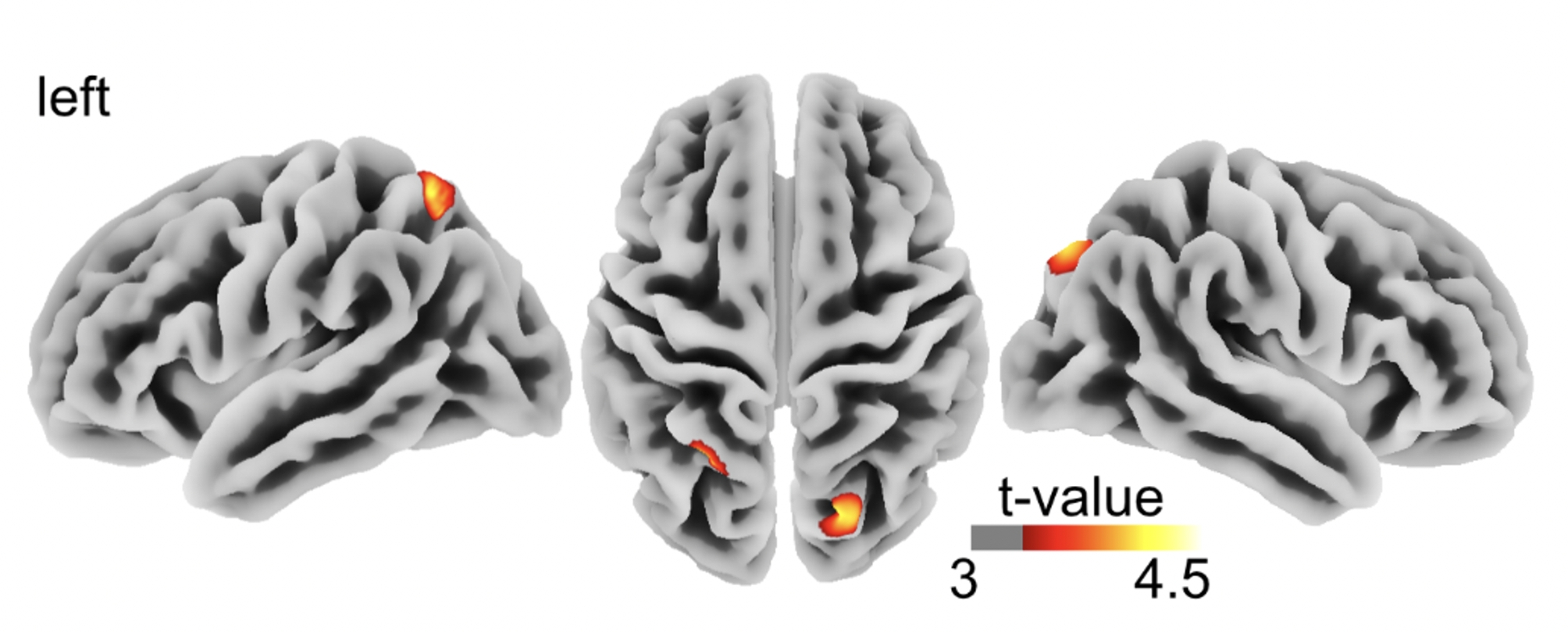
Results of cortical thickness analysis RTHβ patients > controls superimposed on the Freesurfer Average template. Voxels exceeding the statistical threshold of p < 0.05 (FWEc, cluster defining threshold p = 0.001, k = 633) are colour-coded. Significant changes were found in the superior parietal cortex. Colour intensity represents t-values at voxel level

Tractography via diffusion tensor imaging (DTI) revealed significantly higher FA in the corticospinal tract (CST) in RTHß patients (FWEc 0.05, k = 275) (see Fig. 1; Table 1 A). In the RTHβ group, superior parietal cortical thickness was increased bilaterally (FWE(p < 0.05), k = 587) (see Fig. 2 and Table 1B).

**Fig. 3:**
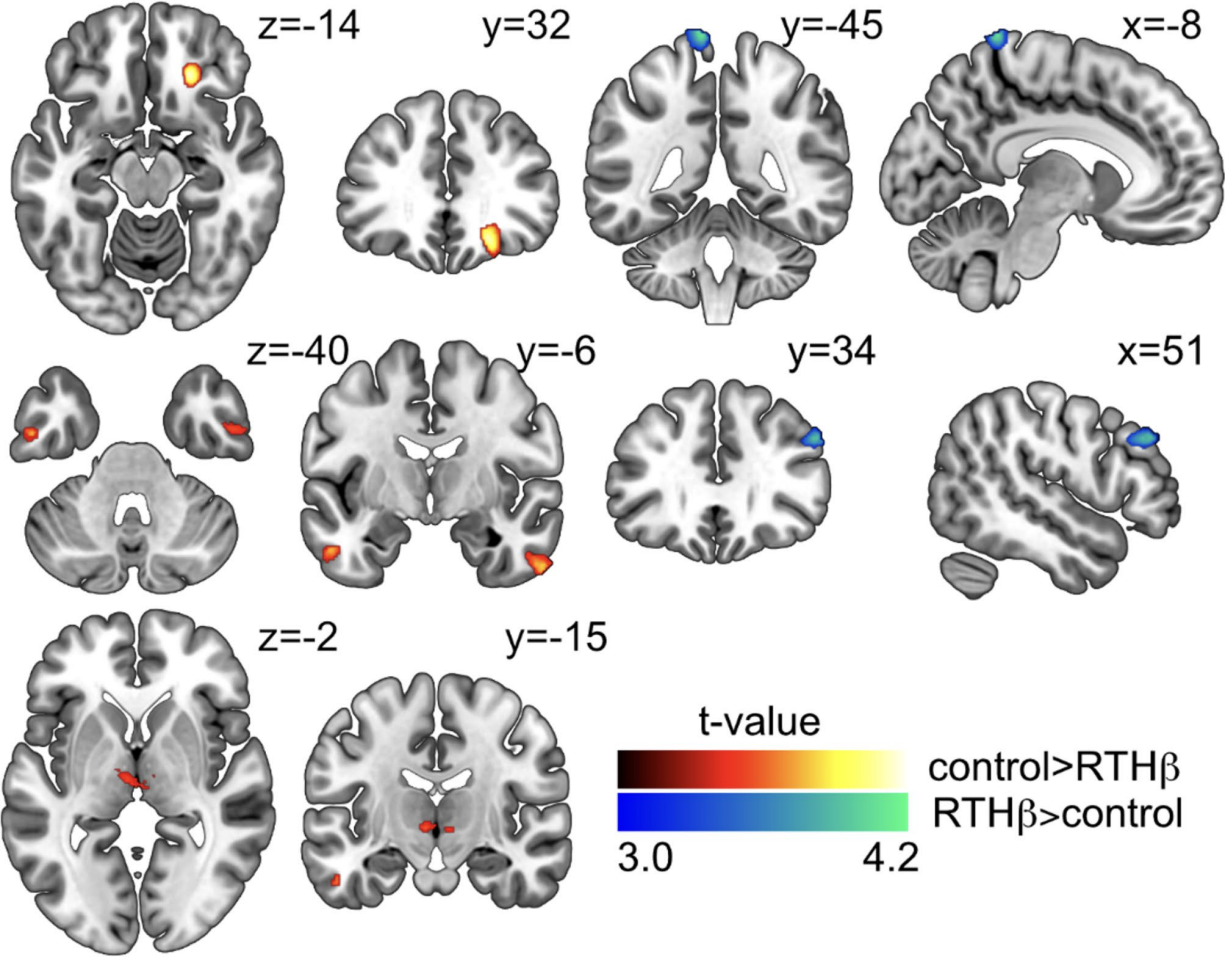
Results of the VBM analysis. Statistical maps are superimposed on a T1-weighted 152-MNI template. Voxels exceeding the statistical threshold of p(unc.) = 0.001 and a cluster threshold of k = 50 are shown in red to yellow (contrast controls > RTHβ patients) and blue to green (RTHβ > control). Colour intensity represents t-values at voxel level

Voxel-based morphometry (VBM) revealed decreased grey matter volume (GMV) bilaterally in the inferior temporal cortex and the thalamus and in the right superior frontal orbital gyrus in RTHβ subjects. Increase in GMV was shown in left precuneus and right middle frontal gyrus in RTHβ subjects. VBM results were based on an uncorrected level (p[unc.] = 0.001, k = 100) (see Fig. 3; Table 1 C).

Analysis of Heschl’s gyri showed no statistical difference when comparing patients and controls (Chi^2^(2) = 4.40; p = 0.11). Similar to published literature [26], we also checked for gender differences in this structural parameter, finding that women in the RTHβ-group had multiple Heschl’s gyri less often than female controls (Chi^2^(2) = 7.6, p = 0.02, see Table 2); for men there was no significant difference in Heschl’s gyri (Chi^2^(2) = 0.47, p = 0.78).

**Table 1:** Peak voxel coordinates

**A) FA maps control > RTHβ**						
location	hemisphere	cluster Size	peak t-value	x	y	z
corticospinal tract	left	567	6.07	110	124	99
corticospinal tract	right	275	4.31	145	137	108
			4.08	139	134	101
**B) Cortical Thickness**						
location	hemisphere	cluster Size	peak t-value	x	y	z
superior parietal cortex	left	720	4.29	18	-89	25
superior parietal cortex	right	633	4.15	-27	-90	32
				-20	-87	26
** C) Voxel based morphometry**						
location	hemisphere	cluster Size	peak t-value	x	y	z
***control > RTH*** **β**						
inferior temporal	left	162	4.08	-50	-12	-28
front sup orb	right	337	4.04	21	32	-14
inferior temporal	right	174	3.80	57	-6	-40
thalamus	left	94	3.58	-6	-15	-2
thalamus	right		3.50	6	-16	-2
***RTH*β >** ***control***						
precuneus	left	247	4.65	-8	-45	78
frontal mid	right	275	4.42	51	34	34

For A: All values cluster corrected FWEc (p = 0.001, k = 275)

For B: All values cluster corrected FWEc = 0.05 (p = 0.001, k = 633). Coordinates and labels are according to the Desikan-Killiany DK40 atlas

For C: All values cluster uncorrected p = 0.001, k = 50. Coordinates in MNI Space, labels according to the AAT3.

**Table 2:** Contingency Table Classification Heschl’s Gyrus

Sex	Group	Heschl			Total
		aa	at	tt	
Female	Control	2	6	2	10
	RTHβ	0	2	8	10
	Total	2	8	10	20
Male	Control	2	3	4	9
	RTHβ	1	4	4	9
	Total	3	7	8	18
Total	Control	4	9	6	19
	RTHβ	1	6	12	19
	Total	5	15	18	38

Frequencies of atypical bilateral (aa), atypical unilateral (at), or typical bilateral (tt) number of Heschl’s gyri. One gyrus is considered as typical (t), more than one as atypical (a)

### Relationship brain structure and attention deficit analysis

**Fig. 4:**
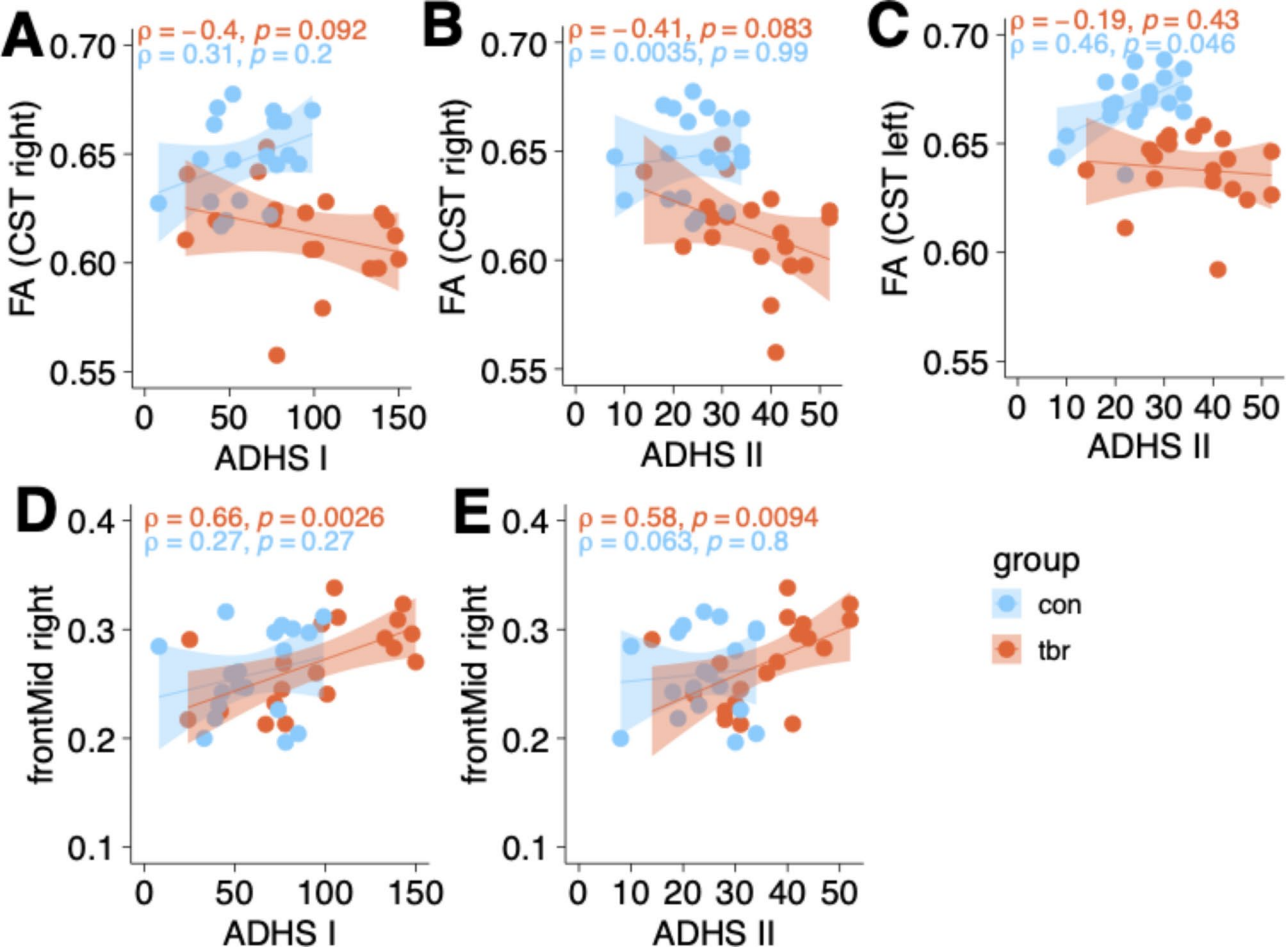
Scatterplots of significant correlations between structural imaging results and ADHD subscales I and II. A-C displays the correlation between mean FA and ADHS I and II scores per group. D-E shows the correlation between mean grey matter at the right midfrontal cluster and ADHS I and II scores per group. Mean FA and grey matter values are extracted from the significant clusters listed in Table 1. See methods for details

With regard to VBM analyses the cluster located in the right midfrontal cortex was significantly correlated with ADHD I (rho = 0.66, p = 0.002) and ADHD II (rho = 0.58, p = 0.009) scores in the RTHβ-group, whereas the control group showed no significant correlation. Furthermore, in the RTHβ-group, decreases in FA values in the right CST were marginally correlated with the ADHD I (rho=-0.4, p = 0.091) and ADHD II (rho = 0.41, p = 0.083) scores. In contrast, FA values in the left CST were positively correlated with ADHD II scores (rho = 0.43, p = 0.046) in the control group (see Fig. 4). Analysis of the cortical thickness ROIs revealed no significant relationship.

## Discussion

As anticipated, RTHß patients showed significant differences in both grey and white matter compared to normal control participants and these changes will be considered in further detail as follows.

**Diffusion tensor imaging** showed that FA in the corticospinal tract differed in RTHß versus control subjects. The corticospinal tract supports motor control of the spinal cord and voluntary movement [37]. It is known that thyroid hormones regulate myelin formation [1], therefore it can be speculated that a changed FA in RTHß can be due to their local hyperthyroid state in the brain influencing white matter tissue and myelin formation. However, the functional relevance of these changes in the corticospinal tract remains to be explored using (for example) transcranial magnetic stimulation and sensitive measures of motor performance.

Changes in white matter have been recorded in hyperthyroid patients with thyroid opthalmopathy [38] and also in patients with Resistance to Thyroid Hormone due to mutations in TRa [39]. Thus, more studies are needed to explore the influence of TH on brain white matter.

**Voxel based morphometry** revealed a decrease of grey matter volume bilaterally in the inferior temporal cortex and the thalamus. The thalamus is a key relay hub, making multiple connections to cortical and subcortical regions. It is also known to play an important role in selective attention, visual and auditory information [40]. The functional significance of these thalamic changes remains to be explored.

The temporal lobe and its associated networks are involved in multiple cognitive domains, including auditory, vision, language, memory, and semantic processing [41].

This structural observation is particularly interesting, since our RTHß patients showed an ADHD-like phenotype, which characteristically involves neuropsychological deficits. In addition, heterozygous RTHb patients exhibit altered retinal photoreceptor function and sensitivity of color perception [15], and this may correlate with the fact that the inferior temporal cortex plays a key part in the visual pathway, including color perception [42].

In a previous study [3] we have analyzed healthy participants with experimentally-induced thyrotoxicosis, revealing an increase of grey matter volume in the posterior part of the cerebellum and a decrease of grey matter volume in the anterior part of the cerebellum. While these observations clearly differ from findings in this study, it has to be kept in mind that the effects of biochemical hyperthyroidism in RTHß patients may be more complex, depending on whether particular brain regions are in a relatively hypothyroid or hyperthyroid state, depending on whether they express mutant TRß or normal TRα. Experimentally-induced thyrotoxicosis also leads to an increased connectivity in temporal lobe structures, caused by an increased connectivity to the cognitive control network [43]. Such increased connectivity supports a role for thyroid hormones in regulating paralimbic structures, with increased degree centrality in the temporal pole being correlated with changes in observed depression scores [43]. This may facilitate prefrontal control over limbic areas, possibly explaining the successful use of thyroid hormones as an augmentation therapy for depression.

**Heschl’s gyrus analysis** showed no difference among groups regarding number of gyri. Whereas one previous study had shown an increased number of gyri in RTHß men [26], this was not replicated by our results. Instead, we found less multiple Heschl’s gyri in RTHß women. We conclude that there is no substantial influence of RTHß on Heschl’s gyrus morphology in our cohort of patients.

**Cortical Thickness** was increased in superior parietal cortex bilaterally in the RTHß group. It is well-known that the parietal cortex is involved in sensory, motor, and cognitive functions, especially regarding space-based and feature-based attention functions and working memory [44]. The parietal cortex is involved in the attention network, parietal cortices generate attention-related modulatory signals and parietal lesions can lead to profound attentional deficits, including visuo-spatial neglect, hereby preventing directing attention contralesionally [45].

ADHD is known to be associated with impairments in attention and with changes in fronto-parietal networks [46], which is relevant because RTHß patients, including participants in the current study, exhibit an ADHD-like phenotype [16–19]. Indeed, increased parietal cortical thickness has also been shown in adult subjects with conventional ADHD [47, 48] whereas reduced cortical thickness was seen in children and adolescents with ADHD [49–51]. Since our study has documented increased parietal cortical thickness in RTHb, it is tempting to postulate that this structural change may be linked to attentional deficits and ADHD-like phenotype in the disorder. With the knowledge that hypothyroidism during development can also affect cortical thickness in various brain regions [25], it is conceivable that resistance to thyroid hormone action which is also a relative hypothyroid state, could have contributed to this morphological change.

Limitations of our study include the relatively small sample size and thus reduced power to detect subtle changes in brain structure. Additionally, the study population was heterogeneous, as RTHb patients from UK were matched with healthy controls from Germany, with a possibility of confounding due to socio-economic and educational differences between the two groups. Nevertheless, we maintain that our study contributes new knowledge about brain structure in this disorder.

## Conclusion

RTHß leads to structural brain changes in cerebral white matter and grey matter. In particular, we found changes in parietal cortical thickness in RTHß. Whether these changes are causally linked to the ADHD-like phenotype seen in RTHß patients, remains to be determined.

## Data Availability

The data that support the findings of this study are available on request from the corresponding author.
